# Post‐Transplant DSA Monitoring After HLAi Kidney Transplantation: Key Time Points

**DOI:** 10.1111/tan.70537

**Published:** 2026-01-08

**Authors:** Ghofran Hijazi, Sunil Daga, Mason Phillpott, David Briggs, Nithya Krishnan, Rob Higgins, Natalia Khovanova

**Affiliations:** ^1^ School of Engineering University of Warwick Coventry UK; ^2^ Leeds Institute of Rhematic and Musculoskeletal Medicine University of Leeds Leeds UK; ^3^ Renal Department Leeds Teaching Hospitals NHS Trust Leeds UK; ^4^ Histocompatibility and Immunogenetics Laboratory NHSBT Birmingham Birmingham UK; ^5^ Renal Department University Hospitals of Coventry and Warwickshire NHS Trust Coventry UK

**Keywords:** classification, DSA, HLA, machine learning, post‐transplant monitoring, transplantation

## Abstract

In HLA‐incompatible transplantation, monitoring donor‐specific antibodies (DSA) after transplantation informs clinical decision‐making and the management of antibody‐mediated rejection. Persistent post‐transplant DSAs are associated with poorer graft survival compared to cases where DSAs resolve. Previously, four distinct early post‐transplant DSA dynamic patterns were identified using a unique dataset with frequent DSA measurements and shown to be associated with AMR and graft outcomes. However, frequent testing is costly, and predicting outcomes using fewer DSA measurements could improve monitoring efficiency. A machine learning classifier based on dynamic time warping was used to identify early post‐transplant DSA patterns from a reduced set of measurements. Individual days and day combinations within the first 50 post‐transplant days were systematically evaluated. The combinations with the highest classification accuracy compared to using all available time points were selected as optimal. This study determined the minimum number of DSA screenings required to accurately classify early DSA dynamics. Measuring DSAs on Days 1, 10, and either 26 or 34 identified the four dynamic patterns with 93.7% accuracy. Including both Days 26 and 34 increased the accuracy to 96.9%. Testing beyond Day 34 did not yield further improvement in classification. Several monitoring regimens commonly used in clinical practice were evaluated, and the proposed approach outperformed all of them. This study identifies an efficient DSA monitoring strategy that can predict antibody behaviour early post‐transplant, enabling timely intervention. It also addresses inconsistencies in current monitoring practices, offering evidence for standardised, data‐driven protocols for more precise and efficient post‐transplant care.

## 1 Introduction

It is well established that preformed anti‐HLA donor‐specific antibodies (DSA), particularly those that persist after transplantation, are strongly associated with both short and long‐term kidney transplant outcomes [1]. Several studies [2, 3, 4] have demonstrated that patients with preformed DSA have a high risk of early antibody‐mediated rejection (AMR) and that patients with persistent posttransplant DSAs can have worse graft survival compared to resolved DSA. While HLA antibody‐incompatible (HLAi) transplantation offers a viable option for sensitised patients [1, 5, 6], it requires complex desensitisation and immunosuppressive protocols to lower pre‐ and post‐transplant DSA levels and reduce the risk of graft failure [7]. Effective posttransplant management, ideally one that is minimally disruptive to patients, depends heavily on understanding the evolution of DSAs after transplantation. However, optimal treatment strategies remain a subject of debate within the transplant community [1]. Therefore, further research into safer approaches to HLAi transplantation, including well‐defined acceptable DSA levels, is essential. Such insights could broaden access to transplantation for sensitised patients and improve long‐term outcomes.

Advances in DSA screening have significantly enhanced our understanding of antibody evolution following transplantation [8, 9]. However, there is still no consensus on optimal DSA monitoring practices [10, 11], and the lack of clear guidance on key monitoring periods continues to hinder effective post‐transplant management, highlighting that optimal methods for predicting graft outcomes are still being developed [12]. It has been demonstrated that DSA fluctuations can be self‐regulated, suggesting that the dynamic behaviour of DSA over time, not just single time‐point measurements, is key to understanding graft outcomes [13, 14, 15].

There are also questions about the routine need for intensified immunosuppression in response to the presence of DSA. Early post‐transplant DSA testing was traditionally performed to aid in the diagnosis of acute AMR and potentially to suggest immediate removal of antibodies. However, our experience shows that early DSA rebound following HLAi is not always associated with clinical rejection, demonstrating that this is not necessarily a pathogenic response in itself. In cases where rejection does occur, it may resolve in the presence of high DSA levels [16], indicating that the priority should be treating the rejection episode itself, rather than targeting the antibody. Instead, we propose that monitoring the dynamic changes in DSA during the early post‐transplant period may offer valuable predictive insights into long‐term graft outcomes.

Research on DSA dynamics after kidney transplantation and the effect of DSA levels on long‐term outcomes is limited, primarily due to the limited resources available for post‐transplant DSA monitoring and the insufficient number of samples for conclusive data analysis. Higgins et al. [14] observed fluctuations in DSA levels within the first month after HLAi transplantation, with peak values occurring within 10–15 days, and an association with early rejection was noted, particularly at higher pre‐treatment and peak post‐transplant levels. DSA levels were also investigated in relation to pregnancy after noticing that women receiving HLAi transplants from their children or spouses are at higher risk of early graft rejection [17]. Several studies have investigated the relationship between systematic DSA monitoring and improved graft outcomes; however, their findings and recommendations vary significantly. Zhang et al. [18] analysed the rise and fall of post‐transplant HLA DSA, reporting more frequent fluctuations during the decay phase and faster DSA clearance in patients with acute AMR. de Castro et al. [19] recommended intensive DSA monitoring primarily for sensitised patients, based on observations of fluctuations within the first month, though their shorter follow‐up may have limited assessment of long‐term effects. Amrouche et al. [20] found that frequent DSA monitoring could improve outcomes in high‐risk patients with elevated median fluorescence intensity (MFI), supporting earlier findings by Kimball [21]. Additionally, Viglietti et al. [22] showed that incorporating post‐transplant DSA measurements into predictive models improves the ability to forecast graft outcomes, suggesting that yearly monitoring may be beneficial.

**FIGURE 1:**
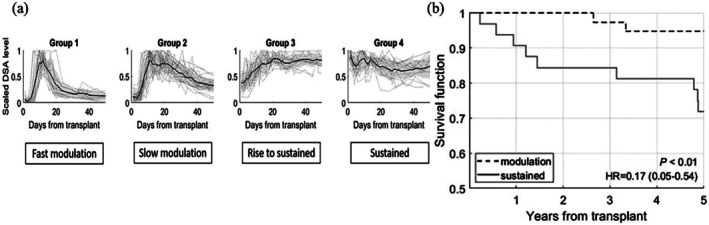
(Taken from study [13]): (a) The four types of DSA dynamical patterns in the early period after HLAi transplantation; (b) Kaplan–Meier analysis comparing the survival rates in modulation (Groups 1 and 2) and sustained (Groups 3 and 4) groups.

Our group has investigated the dynamic changes and the role of the total and HLA‐specific posttransplant DSAs in graft survival [13, 18, 23, 24]. In particular, the most relevant recent publication [13] demonstrates that the DSA dynamics after HLAi exhibit four distinctive patterns (Figure 1a): fast and slow modulated dynamics, and rise to sustained and sustained dynamics. More broadly, these four dynamics were further grouped into two types of responses: modulated and sustained. We showed that modulated responses, when DSA rise and falls within the first 50 days after transplantation, were associated with a significantly better 5‐year death‐censored graft survival (Figure 1b). The research demonstrates the importance of DSA monitoring in the early posttransplant period for improving patient care and long‐term graft outcomes. However, a key limitation of our previous work is that it relied on frequent (almost daily) DSA measurements, which are costly and impractical in clinical settings. The current research builds on these findings, aiming to identify a minimal yet effective set of post‐transplant DSA monitoring points that can accurately predict the dynamic response of DSA. The ultimate goal is to develop evidence‐based DSA screening strategies that are both clinically effective and resource‐efficient, thereby improving post‐transplant care without placing an unnecessary burden on patients or healthcare systems, a key unmet need [25].

## 2 Methods

### 2.1 Patients

We utilised the same dataset as in the Phillpott et al. study [13]. A total of 88 patients were included in the study, who underwent HLAi transplantation between 2003 and 2014 at the University Hospitals Coventry and Warwickshire. The patients were referred from multiple centres in the United Kingdom and the Republic of Ireland for HLAi. There were 13 graft failures [13]. Study approval was obtained from the local ethics committee (CREC‐055/01/03 and 13/WM/0090).

### 2.2 HLA DSA Data

**FIGURE 2:**
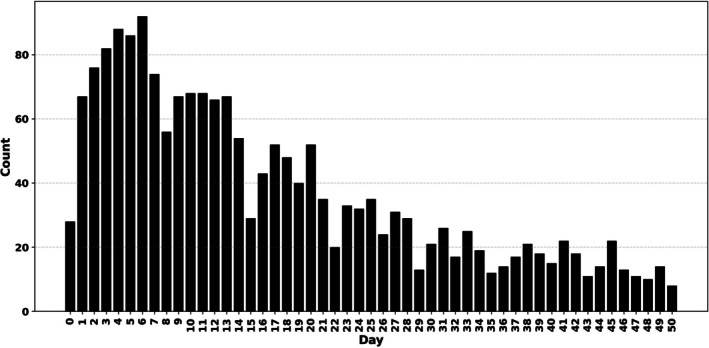
Number of available DSA measurements per day.

Serum samples for post‐transplant DSA analysis were taken almost daily in the first 2 weeks and on alternate days for 3–4 weeks, and sampling was more sparse up to Day 50 (Figure 2). Antibody levels were assessed using the microbead assay manufactured by One Lambda Inc. (Canoga Park, CA, USA) and analysed on the Luminex platform (XMap 200, Austin, TX, USA). The assay measures MFI, a semi‐quantitative measure of antibody level [14]. More details on HLA DSA testing can be found in our previously published work [13]. No specific MFI threshold was used, as our research focuses on the trend. For determination of laboratory positivity, MFI cut‐offs of 1000 (without EDTA) and 2000 (EDTA) were used for screening, and lower MFI threshold if there was clustering of low‐level MFIs against HLAs with the shared epitope. Our patients had between 1 and 7 DSAs targeting different HLA, comprising 211 different DSAs across 88 patients. The sum of individual HLA DSA MFIs was calculated to give a total DSA (total‐DSA) per time point [13]. Missing samples were linearly interpolated.

### 2.3 Time Series Reduction and Predictive Modelling Algorithms

In this analysis, we examined four types of post‐transplant DSA dynamic behaviours (time series) as defined earlier by Phillpott et al. [13] and illustrated in Figure 1: fast modulation, slow modulation, rise to sustained and sustained. The mean time series for each group, shown as dark grey lines in Figure 1a, served as a reference (or centroid) for that group. A distance‐based machine learning classification algorithm using dynamic time wrapping (DTW) [13, 26, 27] was developed to classify the DSA time series into the four patterns from a reduced number of DSA measurements. The algorithm systematically tested individual time points (days) and their combinations to determine which subsets of the time series could best identify the correct DSA pattern. The time point combinations that achieved the highest classification accuracy were identified as optimal for DSA monitoring. In essence, the algorithm compares a partial DSA time series (e.g., comprising one or more DSA measurements) to each group's average trajectory using the DTW distance metric. The pattern is then classified based on which group's average it most closely resembles. Clinical data and patients' characteristics in each group as defined earlier by Phillpott et al. [13] are shown in Table 1.

**TABLE 1:** Characteristics of groups based on clustering.

	Cluster/group					
	0	1	2	3	4	All (*n* = 88)
	No response (*n* = 18)	Fast modulation (*n* = 15)	Slow modulation (*n* = 23)	Rise to sustained (*n* = 16)	Sustained (*n* = 16)	
Age (years, mean)	43.94	44.87	40.17	37.13	39.69	41.10
ESRF (years, mean)	13.08	7.33	10.65	12.16	14.23	11.48
Female (%)	55.56	93.33	78.26	31.25	43.75	61.36
LDKT (%)	94.44	100	95.65	93.75	75	92.05
Previous Tx (%)	72.22	26.67	60.87	81.25	75	63.64
Crossmatch+ (%)	55.56	73.33	69.57	93.75	81.25	73.86
CDC+ (%)	11.11	13.33	17.39	37.5	25	20.45
Out of all CDC+, CDC > 1:2 (%)	0	0	0	100	50	55.56
Pre Tx‐DFPP (%)	66.7	80.0	82.6	93.7	62.5	77.27
Average tDSA value	2062	7023	7105	14813	12674	8735
DSA (number)	2	3	3	3	3	3
Rejection within first 30‐days, %	16.67	80.0	56.52	56.25	18.75	47.73
Post Tx‐DFPP (%)	5.56	33.33	13.04	18.75	6.25	14.77
Lymphocyte‐depleting agent (%)	5.56	40	47.83	50	12.5	31.82
GF‐5 years (%)	11.11	6.67	4.35	31.25	25	14.77

Abbreviations: CDC = complement‐dependent cytoxicity; DFPP = double filtration plasmapheresis; ESRF = end‐stage renal failure; LDKT = living donor kidney transplant; Tx = transplant.

As the baseline (pre‐desensitisation) MFI increased, a more sustained response was observed (*p* < 0.001, Chi‐square test). For the baseline MFI > 5000 (total number of DSA = 58), 3 DSAs (5%) had no response and 37 (64%) had predominantly sustained response (Table 2). Similar findings were observed in MFI titration studies: higher titres were associated with more sustained responses.

**TABLE 2:** Number and percentage of DSA in each dynamic group split according to the baseline (pre‐desensitisation) MFI level.

MFI	Dynamic group (number of DSA, number)						Dynamic group (% DSA)					Broader dynamic (% DSA)		
	0	1	2	3	4	Total	0	1	2	3	4	No response	Modulated	Sustained
< 2500	64	24	21	2	3	114	56	21	18	2	3	56	39	4
2500–5000	5	7	14	6	5	37	14	19	38	16	14	14	57	30
5000–10000	3	10	6	19	10	48	6	21	13	40	21	6	33	60
> 10000	0	0	2	1	7	10	0	0	20	10	70	0	20	80

**FIGURE 3:**
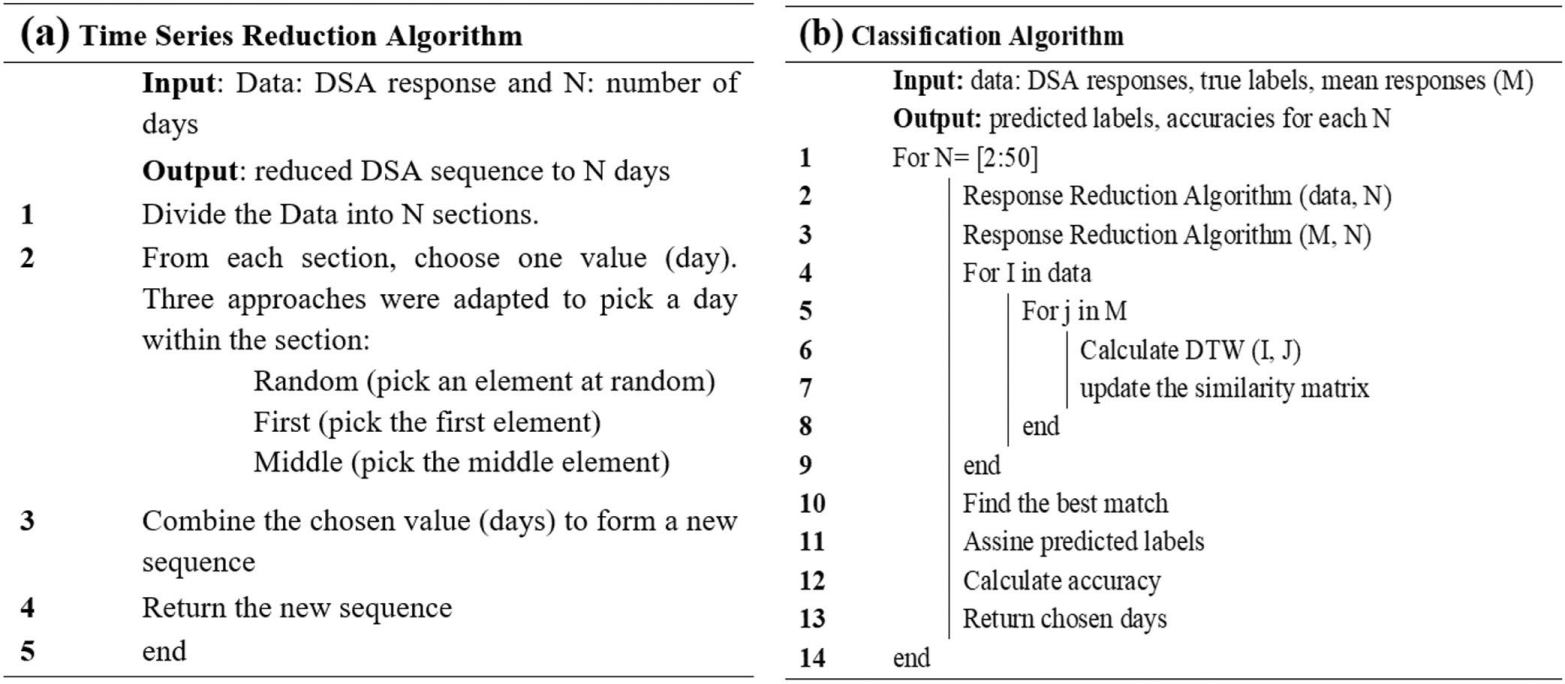
Schematic diagrams illustrate the methodology used to create a reduced DSA time series before classification (a) and classification algorithm (b). I and J are indices used to control how many times a block of an algorithm repeats.

**FIGURE 4:**
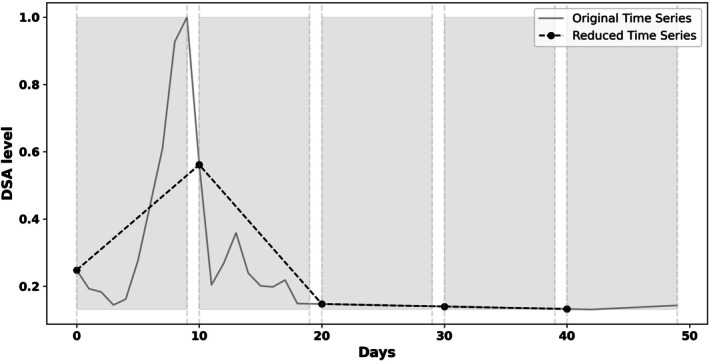
Example of systematically selecting time points for the number of sections *N* = 5.

The time series reduction algorithm, which was developed to create a partial time series with a reduced number of DSA time points *prior to classification*, is shown in Figure 3a. We implemented and compared three different approaches for selecting time points within the *N* sections into which the time series were divided: selecting a point at random within each section, selecting the section's middle point or the section's first point. Figure 4 illustrates the algorithm for *N* = 5, using the random selection approach within each section.

The classification algorithm is summarised in Figure 3b. As described above, the mean response was calculated for each of the four DSA post‐transplant clusters. Both the individual DSA time series and the cluster mean responses were reduced to the same number of time points, N. A similarity matrix was then computed using DTW to measure the distance between each reduced DSA response and the four cluster mean responses. Each reduced time series was assigned to the cluster with the shortest DTW distance. The predicted cluster labels were then compared to the true labels to calculate the classification accuracy. This process was repeated for values of N ranging from 2 to 50, that is, from using just 2 days of DSA monitoring up to the full 50‐day response. The resulting accuracies were compared to determine which time point combinations provided the best classification performance.

### 2.4 Performance Metrics

Classification accuracy was used to compare the results of predictions made using different combinations of test days: $C=\left(N_{\mathrm{cp}}/N_{p}\right)\times 100$, where *N*_*cp*_ is the number of correct predictions and *N*_*p*_ is the number of all predictions.

## 3 Results

Several experiments were conducted using the described methodology under different settings, and the process of identifying the optimal monitoring days was carried out in three stages.

### 3.1 Stage 1: Identifying Initial Monitoring Days

**FIGURE 5:**
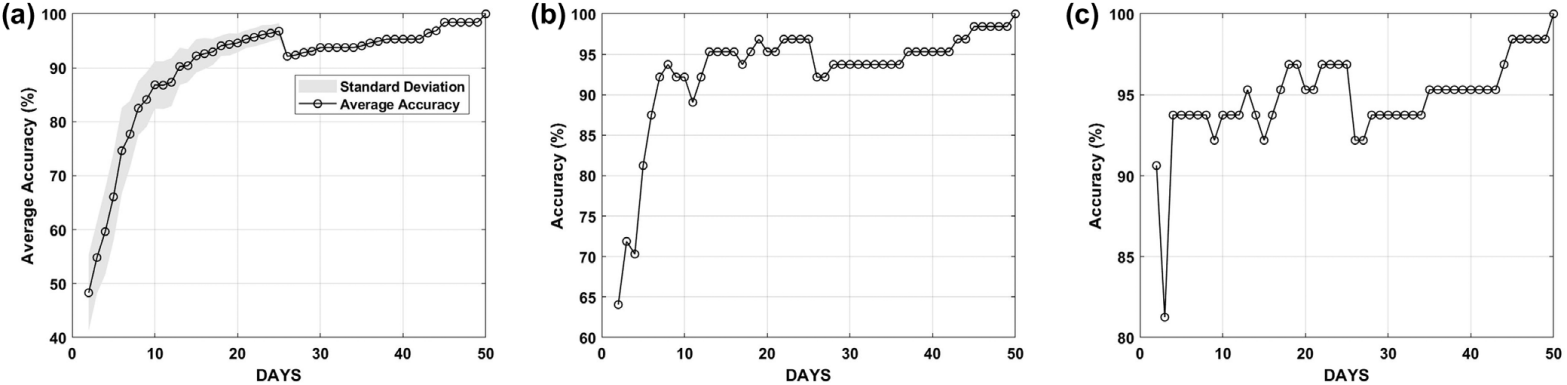
Example of systematically selecting specific days for the number of sections *N* = 5: (a) a point within each section was selected at random, and simulations were repeated 100 times; (b) the middle point was selected, and (c) the first point was chosen from each section.

The number of monitoring days was systematically reduced from 50 to 2 using the three approaches for day selection within sections described earlier: random, the first day of each section and the middle day, and the corresponding classification accuracies *C* were calculated. The results are shown in Figure 5: as expected, increasing the number of days led to higher classification accuracy. However, this trend was not consistent: in some cases, fewer days produced better accuracy than a greater number of days, which was surprising, calling for additional analysis. This further analysis aimed to identify specific days that contributed most to classification performance and should be prioritised in stage 2.

For the random selection setting, the experiment was repeated 100 times, and the mean accuracy was calculated to account for variability. This approach reflects a more realistic clinical scenario, where tests are not always performed at the same time across patients. Figure 5a shows that using just 2 days, one in the first 25 days and one in the last 25 days, can already predict the DSA dynamic pattern with 50% accuracy. Accuracy improved steadily with more days, peaking at 98% when 25 days are considered. As expected, using the full 50‐day time series achieved 100% accuracy. Interestingly, an unexpected drop in accuracy occurred after *N* = 25. Further analysis showed this was due to uneven time division: splitting 50 days into 26 sections resulted in 25 single‐day sections followed by one large section at the end. This distribution skews the coverage, overrepresenting the early period and under‐representing the later days. However, as more days were added and sections became more balanced, accuracy began to recover. A similar trend was observed in the *first* and *middle*‐day selection strategies.

Removing randomness and using fixed points (middle or first day of each section) improved the starting accuracy: with just 2 days (*N* = 2), accuracy reached 64% using the middle‐point strategy (Figure 5b), and around 90.6% using the first‐point strategy (Figure 5c). Further inspection revealed that Days 1 and 26 were commonly selected at this point, indicating their potential importance in classification. These 2 days (Days 1 and 26) were therefore chosen as the starting point for stage 2 considerations.

### 3.2 Stage 2: Identifying Additional Monitoring Days to Improve Accuracy

In this stage, the goal was to determine which additional days, when combined with Days 1 and 26, could further improve classification accuracy while keeping the total number of test days to five or fewer to ensure cost efficiency. The first step was to add a single day between Day 1 and Day 26 and evaluate the effect on accuracy. Day 10 provided the best improvement, raising accuracy to 93.7%. Adding Day 13 or 14 offered a modest increase to around 90%. Day 3 had no effect, while Days 6–9 reduced accuracy to around 82%. Replacing Day 1 with any other day while keeping Days 10 and 26 also reduced accuracy to around 82%, highlighting the importance of testing on Day 1. The importance of testing on Day 1 stems from the nature of the DSA dynamics: modulated patterns typically start at low levels and rise sharply, whereas sustained patterns begin at a high level (Figure 1). Therefore, the absence of a test from Day 1 would make it harder to distinguish between these patterns, leading to misclassification.

Next, an additional day was tested between Days 27 and 37. Adding Day 34 resulted in the greatest improvement, raising accuracy to 96.9%. Other days had only minimal or no positive effect. Testing beyond Day 34 yielded no further improvement either. Further analysis of combinations revealed that Days 1, 10 and 26 (or 34) gave 93.7% accuracy. Including both Days 26 and 34 together provided the best accuracy of 96.9%.

In summary, monitoring DSA on Days 1, 10 and 26 (or 34) can classify the dynamic pattern with 93.7% accuracy. Including both Days 26 and 34 further improves accuracy to 96.9%. No additional benefit was observed from testing beyond Day 34.

### 3.3 Stage 3: Comparison With Clinical Protocols

To evaluate the effectiveness of the proposed monitoring schedule, we compared it against DSA testing regimens commonly used in clinical practice. A literature review was conducted to identify typical DSA monitoring time points used in clinical settings. Testing regimens that included DSA levels on days not available in our dataset, for example, beyond 50 days post‐transplant, were excluded. The testing regimen from the remaining published work [14, 28], was passed into our classification algorithm using our data. The resulting accuracies were calculated and compared. The comparison results are presented in Table 3. It shows that our proposed schedule (settings F and G) outperforms all other regimens (A–E).

**TABLE 3:** Monitoring protocols used by different medical centres.

Reported regimen	Post‐transplant DSA monitoring days	Predictive accuracy %
A [14]	1–14,17,18,20,22,27,30	95.3
B [26]	1,8,15,22	84.4
C [29]	1,7,30	87.5
D [19]	4,7,14,30	84.4
E [30]	1–3,7,14,21,28	92.2
Our setting F (Figure 5c)	1,10,26 or 34	93.7
Our setting G (Figure 5c)	1,10,26,34	96.9

## 4 Discussion

This study identified the minimum number of post‐transplant DSA screenings required to accurately classify DSA dynamics into four previously defined patterns following HLAi kidney transplantation. Using a machine learning classification algorithm based on dynamic time warping, the study found that DSA measurements on Days 1, 10 and either 26 or 34 post‐transplant achieved 93.7% classification accuracy. Including both Days 26 and 34 increased accuracy to 96.9%, while additional testing beyond Day 34 did not improve performance.

The behaviour of DSA following HLAi depends on several factors, primarily the balance between regulatory and effector immune responses, as well as the maturity stage of the immune system. Long‐lasting plasma cells will produce background DSA before transplantation and following previous immune responses during sensitisation. Upon re‐exposure to the same antigen, a recall immune response may be triggered, leading to rapid synthesis of DSA [17]. This can occur via T cell‐independent memory responses, the proliferation of B cells into short‐lived plasma cells and/or through T cell‐dependent mechanisms involving the migration of memory B cells and their interaction with follicular helper T cells, resulting in the production of long‐lasting plasma cells. These processes can alter both the quantity and quality of DSA through subclass switching and affinity maturation.

These recall mechanisms, both T‐cell dependent and independent, can occur in addition to the baseline DSA production driven by long‐lived plasma cells. Why some patients exhibit only short‐lived transient responses (Group 1, Figure 1) while others show sustained DSA production (Group 3, Figure 1) remains a matter of significant interest. It is likely to be explained by the heterogeneity of patient‐specific factors [13, 31], the nature of the initial sensitisation events [17], and variable responses to immunosuppressive medications. In certain cohorts, particularly younger male patients and those sensitised by prior transplants, the immune response may be at a more advanced stage of affinity maturation, as evidenced by predominantly sustained responses (Group 3, Figure 1) post‐transplantation [13]. This is further supported by the presence of complement‐fixing DSA, often associated with high‐affinity antibodies, detected before transplantation and linked to a modulatory or sustained response [32]. We have previously described a cohort of patients with no DSA rise following HLAi [13], and this observation has been confirmed by recent Imlifidase studies [30, 33, 34], which suggest the existence of patients who develop no measurable DSA response post‐transplant. Equally, sustained DSA production has been associated with recurrent rejection episodes and progression to chronic AMR [35]. Thus, the heterogeneity in immune profiles before transplantation may explain the heterogeneous post‐transplant DSA dynamics observed across patients. Importantly, the DSA dynamic within the first month post‐transplant reflects the underlying immune risk, likely defined by both memory quality and long‐lived plasma cells, and has significant implications for long‐term graft survival [36]. Characterising these early dynamic patterns offers valuable clinical insight and may guide more personalised patient management.

This study defined a minimal set of tests necessary to characterise early DSA dynamics as a surveillance strategy, addressing a key unmet need identified by the ESOT consensus group [25]. These findings have important implications for post‐transplant care and suggest a framework on which additional measurements can be added for clinical reasons, such as when assessing the response to treatment for AMR. Compared to other references in Table 1, our classification did not identify Day‐7 as a key time point for assessing overall DSA dynamics. However, we acknowledge that this may delay recognition of a rebound in Group 1 patients who are not showing clinical signs of concern, as it would only be detected on Day 10. In such cases, earlier identification of DSA rebound could prompt clinicians to consider an earlier biopsy, even in the absence of clinical symptoms (whether protocol‐ or DSA‐driven). Nevertheless, in our cohort, we did not manage a rebound in the absence of clinical concerns; thus, Day‐7 measurement may have limited clinical significance. Similarly, the number of time point measurements can also be guided by the pre‐transplantation MFI as we observed higher no responses if baseline MFI was below 2500 (Table 2).

By identifying the most informative time points for DSA monitoring, clinicians can streamline follow‐up DSA monitoring strategies, improving efficiency and cost‐effectiveness. Combined with our previous findings linking sustained DSA responses to poorer graft outcomes [13], this approach may enable early identification of patients at increased risk of graft failure within the first 50 days, allowing for timely interventions and improved long‐term transplantation outcomes.

Additionally, this study addresses a key gap in current practice, where DSA monitoring schedules are often inconsistent and lack clear, evidence‐based guidelines [25]. The findings contribute valuable evidence to support the development of standardised, data‐driven monitoring protocols, aiming to improve both the precision and efficiency of post‐transplant care. Future research should explore the generalisability of these findings in larger and more diverse patient cohorts, opening the door to personalised DSA monitoring strategies tailored to individual patient profiles.

## Author Contributions

**G.H.:** study design, methodology and analysis, first draft (joint), funding. **S.D.:** data collection, study design, analysis, editing, PhD supervision. **M.P.:** data collection, editing. **R.H.:** data collection, design, editing. **N.Kr.:** data collection, editing. **D.B.:** data collection, design, editing. **N.Kh.:** study design, methodology and analysis, first draft (joint), funding, editing and PhD supervision.

## Funding

We acknowledge EPSRC UK grants (EP/K02504X/1 and EP/W524645/1), and UHCW NHS Trust—Renal Transplant research funds for funding the project. S.D. is supported by the National Institute for Health Research HealthTech Research Centre for Accelerated Surgical care.

## Conflicts of Interest

The authors declare no conflicts of interest.

## Data Availability

The datasets presented in this article are not readily available because they are available on request and for collaborative research only. Requests to access the datasets should be directed to Natalia Khovanova at n.khovanova@warwick.ac.uk.
